# Risk Evaluation of Pathogenic Intestinal Protozoa Infection Among Laboratory Macaques, Animal Facility Workers, and Nearby Villagers From One Health Perspective

**DOI:** 10.3389/fvets.2021.696568

**Published:** 2021-09-29

**Authors:** Jian Li, Yijing Ren, Haiying Chen, Weiyi Huang, Xinyu Feng, Wei Hu

**Affiliations:** ^1^Department of Pathogen Biology, Basic Medical College, Guangxi Traditional Chinese Medical University, Nanning, China; ^2^Department of Infectious Diseases, Huashan Hospital, State Key Laboratory of Genetic Engineering, Ministry of Education Key Laboratory for Biodiversity Science and Ecological Engineering, Ministry of Education Key Laboratory of Contemporary Anthropology, School of Life Sciences, Fudan University, Shanghai, China; ^3^Department of Veterinary Preventive Medicine, College of Animal Science and Technology, Guangxi University, Nanning, China; ^4^National Institute of Parasitic Diseases, Chinese Center for Disease Control and Prevention (Chinese Center for Tropical Diseases Research), Shanghai, China; ^5^NHC Key Laboratory of Parasite and Vector Biology, Shanghai, China; ^6^WHO Collaborating Centre for Tropical Diseases, Shanghai, China; ^7^National Center for International Research on Tropical Diseases, Shanghai, China; ^8^Joint Research Laboratory of Genetics and Ecology on Parasite-Host Interaction, Chinese Center for Disease Control and Prevention & Fudan University, Shanghai, China; ^9^School of Global Health, Chinese Center for Tropical Diseases Research, Shanghai Jiao Tong University School of Medicine, Shanghai, China; ^10^One Health Center, Shanghai Jiao Tong University-The University of Edinburgh, Shanghai, China; ^11^Department of Biology, College of Life Sciences, Inner Mongolia University, Hohhot, China

**Keywords:** intestinal protozoan, *Macaca mulatta*, epidemiology, genotype, haplotype, one health

## Abstract

**Background:** Previous epidemiological studies have confirmed non-human primates (NHPs) as reservoirs for *Cryptosporidium* spp. , *Giardia intestinalis*, and *Enterocytozoon bieneusi*. It highlights the possibility of interspecies transmission between humans and macaques in laboratory animal facilities. This study aimed to investigate the prevalence of pathogenic intestinal protozoan infections in macaques and humans and to determine the risk of cross-species transmission from One Health view.

**Materials and Methods:** A total of 360 fecal samples, including 310 from the four *Macaca mulatta* groups, 25 from the facility workers in a laboratory animal facility, and 25 from the villagers nearby in Yongfu country, southeast China, were collected. Nested PCR assays were done for detecting protozoan pathogens from all the specimens. Furthermore, potential risk factors (gender, age, and direct contact) on the occurrence of intestinal protozoa infection among different sub-groups were evaluated. A phylogenetic and haplotype network analysis was conducted to examine the genetic structure and shared patterns of *E. bieneusi* and *Cyclospora cayetanensis*.

**Results:** The pathogenic intestinal protozoa were detected in both human and macaque fecal samples. A total of 134 (37.2%) samples were tested positive, which included 113 (36.4%) macaques, 14 (56.0%) facility workers, and 7 (28.0%) villagers, respectively. There was no significant difference in four intestinal protozoa infections between facility workers and villagers (χ^2^ = 2.4, *P* > 0.05). However, the positive rate of pathogenic intestinal protozoa in the facility workers, who had direct contact with macaques, was significantly higher [odds ratio (OR) = 0.31, 95% confidence interval (CI): 0.09–1.00, *P* < 0.05).Thirty-three *ITS* genotypes of *E. bieneusi* were identified, including five known genotypes (PigEBITS7, Peru8, Henan V, D, and CM1) and six novel genotypes (MEB1–6). Seven haplotypes were identified in the network analysis from *C. cayetanensis*-positive samples. Meanwhile, a phylogenetic and haplotype analysis confirmed the presence of zoonotic subtypes in NHPs and humans.

**Conclusion:** The data collected from this study confirmed a high prevalence of intestinal protozoan infection in humans and macaques. These results warrant workers of such facilities and residents to limit contact with infected animals in order to minimize related health risks. The need for comprehensive strategies to mitigate the risk of zoonotic transmission, especially from a One Health perspective, is recommended.

## Introduction

Several pathogenic intestinal protozoa, including *Enterocytozoon bieneusi, Cyclospora cayetanensis, Cryptosporidium* spp., and *Giardia intestinalis*, are the causative agents of gastrointestinal diseases with a primary clinical symptom of diarrhea in humans and animals worldwide (1, 2). Many of these pathogens can be transmitted to humans by direct contact or the ingestion of contaminated food and water and are therefore usually defined as foodborne or waterborne parasites (3–5). Elders, children, and immunocompromised individuals are more susceptible to these pathogenic intestinal protozoa (6–8).

The application of molecular tools to identify species, genotypes, and sub-genotypes of the intestinal protozoa has dramatically improved their classification, transmission, and virulence. For example, the *ITS2* gene-based identification of the genotypes facilitates the taxonomy of *E. bieneusi* and identified potential zoonotic species (9, 10). Similarly, employing *Giardia duodenalis* genes (genetic loci), only the assemblages A and B of *Giardia* spp. were found to be zoonotic (4). Furthermore, the *60-kDa glycoprotein* (*gp60*) was used in subtyping the *Cryptosporidium* spp., which helps to identify the hyper-transmissible IIaA15G2R1 subtype in *Cryptosporidium parvum* and the virulent IbA10G2 subtype in *Cryptosporidium hominis* (11). With the increased usage and the integration of molecular tools, an improved understanding of zoonotic transmission will aid research and prevent infection in the future.

The studies of life sciences require many laboratory animals; among which, non-human primates (NHPs) are the essential living experimental animals by similarity in their physiological characteristics to humans (12). China has become a significant supplier of NHPs since the 1970s after India banned their export. More than 39 commercial NHP facilities have been established, mainly raising cynomolgus macaques (*Macaca fascicularis*) and rhesus macaques (*Macaca mulatta*). Therefore, there is a risk of interspecies transmission of pathogens between humans and macaques. For example, macacine herpesvirus 1 (MaHV1; B virus) can lead to fatal encephalitis in humans if they encounter animal-related exposures such as being bitten or scratched by an infected macaque monkey (13). Recently, a simian malaria parasite, *Plasmodium cynomolgi*, has made the jump to infect humans and become zoonotic (14) naturally. In the context of the COVID-19 pandemic, One Health provides a unique perspective and primary care on the health of humans and the health of animals and closely connected our shared environment (14). The present study was conducted to detect and characterize intestinal protozoa (*E. bieneusi, Cyclospora* spp., *Cryptosporidium* spp., and *G. intestinalis*) in rhesus macaques, facility workers, and residents in nearby villages in order to better inform the potential health risk for both human and animal, which would have great public health significance.

## Materials and Methods

### Ethics Statement

This study was conducted following the Regulations for the Administration of Laboratory Animal in China. The research protocol was reviewed and approved by the Ethics Committee of Fudan University. Before the sampling, appropriate permission was obtained from the director of animals and properties.

### Sample Collection

The commercial laboratory animal facility is located in Guangxi Autonomous Region in China. Mountains surround it on three sides, and a village called Qinmu is next to the fourth side. The farmhouse contained 24 door-to-door independent feeding rooms in two rows. The separate rooms are about 15 m^2^ with an iron mesh of about 1 m above the ground. The facility was established in 2001, where over 5,000 animals were reared at the time of sampling.

The macaque fecal samples, taken from a total of 310 rhesus macaques (*M. mulatta*) in August 2015, were divided into four groups according to rearing mode and defined as follows: (1) the breeding macaques (BreM) group contained mainly sexually mature females. (2) The fattening macaques (FatM) group included monkeys of 6 months−2 years old. (3) The teenage macaques (TeeM) were composed of monkeys of 2–3.5 years of age. They were primary commercial monkeys. (4) The adult male macaque group (AduM) includes male aged over 3.5 years old. A single fresh fecal sample was collected from each macaque immediately following the defecation in a labeled and sterile fecal container. Additionally, 25 stool samples were collected from the workers in the facility, and 25 stools samples were from the villagers in Qinmu country. Each piece (about 10 g) was collected into a plastic container and labeled. The samples were stored in 2.5% (*w*/*v*) potassium dichromate at 4°C before DNA extraction.

### DNA Extraction and PCR Amplification

Approximately 200 mg/ml of each sample was used for the extraction of parasite genomic DNA using E.Z.N.A.R^®^ Stool DNA Kit (Omega Biotek Inc., Norcross, GA, USA), according to the manufacturer's instructions. The extracted DNA samples were stored at −20°C until PCR amplification.

The nested PCR assay targeting the sequence of the *ITS* rRNA gene was used to amplify *C. cayetanensis* from specimens. The specific primer pairs were designed as described by Sulaiman et al. (15). The genotypes were named based on an established nomenclature system (9). A *C. cayetanensis*-specific nested PCR of the *SSU* rRNA gene was performed on macaque samples as previously published (16). Nested PCR assay was also done for detecting *Cryptosporidium* spp. and *G. intestinalis* by amplifying *SSU* rRNA (17) and the *SSU* rRNA gene (18), respectively.

All the secondary PCR products, which were positive and corresponding to the protozoan parasites, were directly sequenced using a set of primers used for secondary PCR after being purified. The obtained sequences were edited using DNASTAR software (www.dnastar.com/software/lasergene/) and aligned using ClustalX (http://www.clustal.org/clustal2/). The genotypes of *E. bieneusi*, acquired in the present study, were given the published name if they were identical to the known genotypes in GenBank. New genotypes were named according to the established nomenclature system based on 243 bp of the *ITS* gene region of *E. bieneusi* (9). The genotypes of *C. cayetanensis* are based on 445 bp of the *SSU rRNA* gene. The species of *Cryptosporidium* spp. and *Giardia* spp. were identified using BLAST (blast.ncbi.nlm.nih.gov/Blast.cgi) with the highest identity. The nucleotide sequences obtained in the present study have been deposited in GenBank database under the accession numbers MN890022-MN890026 (*E. bieneusi*), MN893885-MN893894 (*C. cayetanensis*), MN893902-MN893909 (*Cryptosporidium andersoni*), MN894004-MN894009 (*C. parvum*), and MN897749-MN897750 (*G. intestinalis*).

### Haplotype Network Analysis and Phylogenetic Reconstruction

To visualize the genetic structure characteristics of the intestinal protozoa, we included the *E. bieneusi ITS2* sequences dataset, containing all the zoonosis genotypes and NHP genotypes (15, 19–21). We also included *C. cayetanensis* partial *SSU rRNA* sequences dataset, containing the genotypes of human and non-human primates (22–25). The multiple sequence alignment of both datasets was performed using ClustalW implemented in MEGAX (www.megasoftware.net) (26). The unique haplotypes were identified in DnaSP27 prior to the network analysis. The network reconstruction was carried out using a statistical parsimony network analysis with the connection probability set to 95%. The haplotype networks were analyzed in the TCS Networks (27), and the frequencies of specimens were displayed in PopART (28).

Phylogenetic trees were constructed using the neighbor-joining (NJ) method in MEGAX (26). Haplotypes with frequencies >1% were selected for analysis. The genotype PtEbIX (DQ885585), isolated from a dog, was used as an out-group for the construction of the phylogenetic trees of *E. bieneusi*. For the construction of phylogenetic tree of *C. cayetanensis*, the *Eimeria perforans* (EF694017), *Eimeria langebarteli* (AF311640), *Eimeria catronensis* (AF324213), and *Eimeria meleagrimitis* (AF041437) were used as out-groups. The phylogeny was tested with 1,000 bootstrap replicates, using the Kimura two-parameter model as a nucleotide substitution model and gamma distribution as rates among sites.

### Statistical Analysis

Chi-square tests were used to calculate the *P-*value with odds ratios (OR) and 95% confidence intervals (CI) to determine the prevalence of intestinal protozoan infections among different groups. All statistical significance was set to a value of *P* < 0.05. GraphPad Prism (Version 8.02, GraphPad Software Inc.) was used for the analysis.

## Results

### Prevalence of Pathogenic Intestinal Protozoa in Macaques and Humans

The overall intestinal protozoa prevalence of parasitic infection was 37.2%. There were 28.7% (89/310) of macaques, 56.0% (14/25) of facility workers, and 28.0% (7/25) of villagers infected with one or more protozoa (*E. bieneusi, C. cayetanensis, Cryptosporidium* spp., and *G. intestinalis*).

A total of 31 (8.6%) of the samples were *E. bieneusi* positive (7, 6.5% in humans; 24, 7.7% in macaque). Five known genotypes (PigEBITS7, Peru8, Henan V, D, and CM1) and six novel genotypes (MEB1–6) were found. *C. cayetanensis* was detected in 5 (10.0%) human samples and 23 (7.4%) macaque samples. The highest prevalence of *E. bieneusi* and *C. cayetanensis* was found in facility workers (Table 1). Three species of *Cryptosporidium* spp. were detected, which included *C. parvum* (7.5%), *C. andersoni* (5.3%), and *C. hominis* (1.4%). *C. parvum* was only found in facility workers (16.0%), but the other two species were found in the villagers (3.4% and 8.6%, respectively). The *G. intestinalis* was observed in 7.2% (26/360) of the fecal samples tested. The coinfection rate was relatively low, occurring at a rate of 36.1% (co-occurrence of *C. cayetanensis* and *G. intestinalis* accounted for 30.7%).

**Table 1:** Occurrence of *Enterocytozoon bieneusi, Cyclospora cayetanensis, Cryptosporidium* spp., and *Giardia duodenalis* in humans and macaques.

	**Intestinal protozoa**	***Enterocytozoon bieneusi***		***Cyclospora cayetanensis***	***Cryptosporidium*** **spp**.			***Giardia intestinalis***
	**Group**	**No. of positive/No. of examined (%)**	**Genotype (*n*)**	**No. of positive/No. of examined (%)**	***C. parvum***	***C. andersoni***	***C. hominis***	**No. of positive/No. of examined (%)**
					**No. of positive/No. of examined (%)**	**No. of positive/No. of examined (%)**	**No. of positive/No. of examined (%)**	
Humans	Facility worker	6/25 (24.0)	D (3), CM1 (2), MEB5(1)	4/25 (16.0)	4/25 (16.0)	0/25 (0.0)	0/25 (0.0)	2/25 (8.0)
	Villager	1/25 (4.0)	D(1)	1/25 (4.0)	0/25 (0.0)	4/25 (16.0)	0/25 (0.0)	2/25 (8.0)
	Sub-total	7/50 (6.5)		5/50 (10.0)	4/50 (8.0)	4/50 (8.0)	0/50 (0.0)	4/50 (8.0)
Macaques	Breeding	5/99 (5.1)	D (1), CM1 (3), MEB2 (1)	6/99 (6.0)	4/99 (4.0)	7/99 (7.1)	1/99 (1.0)	5/99 (5. 1)
	Fattening	10/58 (17.2)	D (1), CM1 (4), PigEBITS7 (1), MEB3 (2), MEB4 (1), MEB6 (1)	6/58 (10.3)	5/58 (8.6)	2/58 (3.4)	5/58 (8.6)	5/58 (8.6)
	Teenage	1/79 (1.3)	CM1 (1)	11/79 (13.9)	7/79 (9.0)	5/79 (6.3)	0/79 (0.0)	5/79 (6.3)
	Adult male	8/74 (10.8)	D (2), CM1 (1), Peru8 (2), Henan V (1), MEB1 (1), MEB3 (1)	0/74 (0.0)	7/74 (9.5)	1/74 (1.4)	0/74 (0.0)	7/74 (9.5)
	Sub-total	24/310 (7.7)		23/310 (7.4)	23/310 (7.4)	15/310 (4.8)	6/310 (1.9)	22/310 (7.1)
Total		31/360 (8.6)		28/360 (7.8)	27/360 (7.5)	19/360 (5.3)	6/360 (1.6)	26/360 (7.2)

### Factors Associated With the Prevalence of Pathogenic Intestinal Protozoa

The positive rate of pathogenic intestinal protozoa in the facility workers, who were in contact with macaques, was significantly higher than that in the villagers (OR = 0.31, 95% CI: 0.09–1.00, *P* < 0.05). However, no significant difference was detected in each kind of intestinal protozoa infection rate between the two groups (*E. bieneusi*: χ^2^ = 2.6, *P* > 0.05; *C. cayetanensis:* χ^2^ = 0.9, *P* > 0.05; *Cryptosporidium* spp.: χ^2^ = 0.0, *P* > 0.05; and *G. intestinalis:* χ^2^ = 0.0, *P* > 0.05). Among the different groups in macaques, Pearson's chi-squared test showed no statistically significant difference between the male and female macaques (BreM vs. AduM, χ^2^ = 0.2, *P* > 0.05). There was a significant difference in infection rate between macaque of different age groups. Notably, the infection rate decreased with age, and the infection rate in the FatM group aged between 6 months and 2 years was almost two times higher than that in the AduM group aged above 3.5 years (48.3% vs 23.0%; OR = 0.32, 95% CI: 0.15–0.68, *P* < 0.05). Additionally, no significant difference in four intestinal protozoa infection rates was identified between different subgroups, except in TeeM (χ^2^ = 12.3, *P* < 0.05) (Table 2).

**Table 2:** Prevalence and risk factors for intestinal protozoan infections in humans and macaques.

**Variable**	**Groups**	**No. tested**	**No. positive**	**% (95% CI)**	**OR (95% CI)**	***P-*value**
Occupation	Villagers	25	7	28.00 (10.40–45.60)		
	Workers	25	14	56.00 (36.54–75.46)	0.31 (0.09–1.00)	*P* < 0.05
Human-macaques contact	Macaques	310	113	36.45 (31.09–41.81)		
	Workers	25	14	56.00 (36.54–75.46)	0.45 (0.21–1.00)	*P* > 0.05
Macaques	BreM (vs. AduM)	99	20	20.20 (12.29–28.11)	1.18 (0.57–2.36)	*P* > 0.05
	FatM (vs. TeeM)	58	28	48.28 (35.41–61.14)	0.47 (0.23–0.95)	*P* < 0.05
	TeeM (vs. AduM)	79	24	30.38 (20.24–40.52)	0.68 (0.33–1.41)	*P* > 0.05
	AduM (vs. FatM)	74	17	22.97 (13.39–2.56)	0.32 (0.15–0.68)	*P* < 0.05

### Phylogenetic Relationship and Haplotype Analysis of *E. bieneusi*

**Figure 1:**
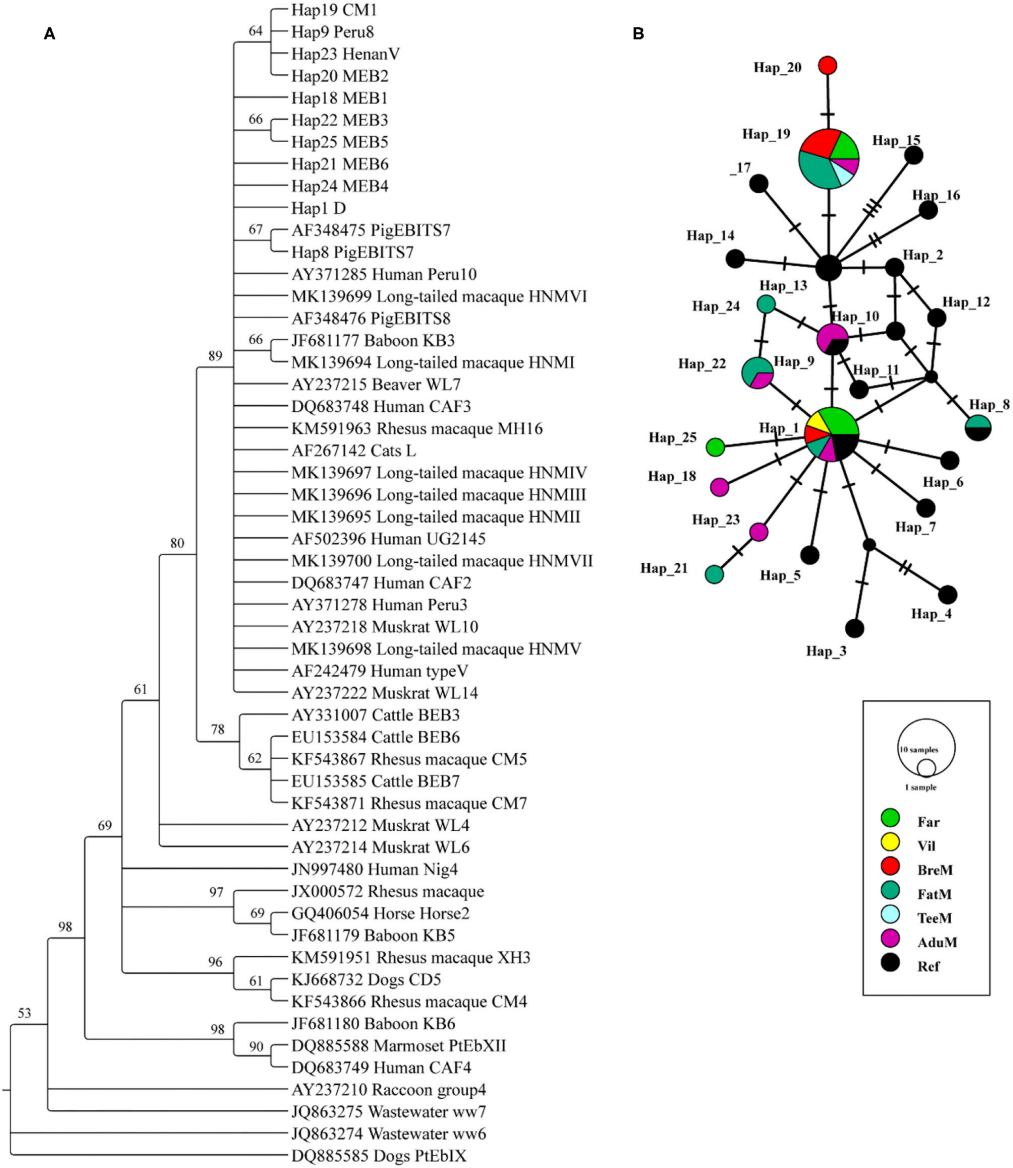
Phylogenetic tree constructed using neighbor-joining (NJ) method and haplotype analysis network of *Enterocytozoon bieneusi* based on ~243 bp sequence of the internal transcribed spacer 2 (*ITS2*) region. **(A)** The phylogenetic analysis tree was constructed using the Kimura two-parameter model with 100 bootstrap replications. Eleven haplotypes were used in tree construction. **(B)** All the 31 sequences in this study and 19 published sequences in group 1 cluster were used to generate haplotype data. The network was constructed using TCS Network algorithm. Various colors represent different groups and the sizes of the circle represent the numbers of haplotypes. Far, facility workers; Vil, villagers; BreM, breeding macaques; FatM, fattening macaques; TeeM, teenage macaques; AduM, adult male macaques; Ref, reference published sequences.

A total of 24 and 7 *ITS* sequences were obtained from the *E. bieneusi* of macaques and humans, respectively. All the sequences were trimmed to 243 bp and aligned to the reference sequences. The phylogenetic tree was reconstructed using the 10 unique haplotypes and main genotypes of *E. bieneusi* from previous studies (19, 21) (Figure 1A). All the haplotypes identified in this study belonged to potential zoonotic species. Twenty-five haplotypes were shown in the networks, and 10 unique haplotypes were collapsed. Three haplotypes (Hap1, Hap19, and Hap25) were detected from humans, while the Hap1 is the most common haplotype, accounting for 57.1% of the total known genotype D. A total of nine haplotypes (Hap1, Hap8, Hap9, and Hap18–24) were detected in macaques; among them, Hap19 was the most common haplotype, accounting for 37.5% of the known genotype CM1. Five unique new haplotypes were found from macaques, and one unique new haplotype was found from humans (Supplementary Table S1; Figure 1B).

### Phylogenetic Relationship and Haplotype Analysis of *C. cayetanensis*

**Figure 2:**
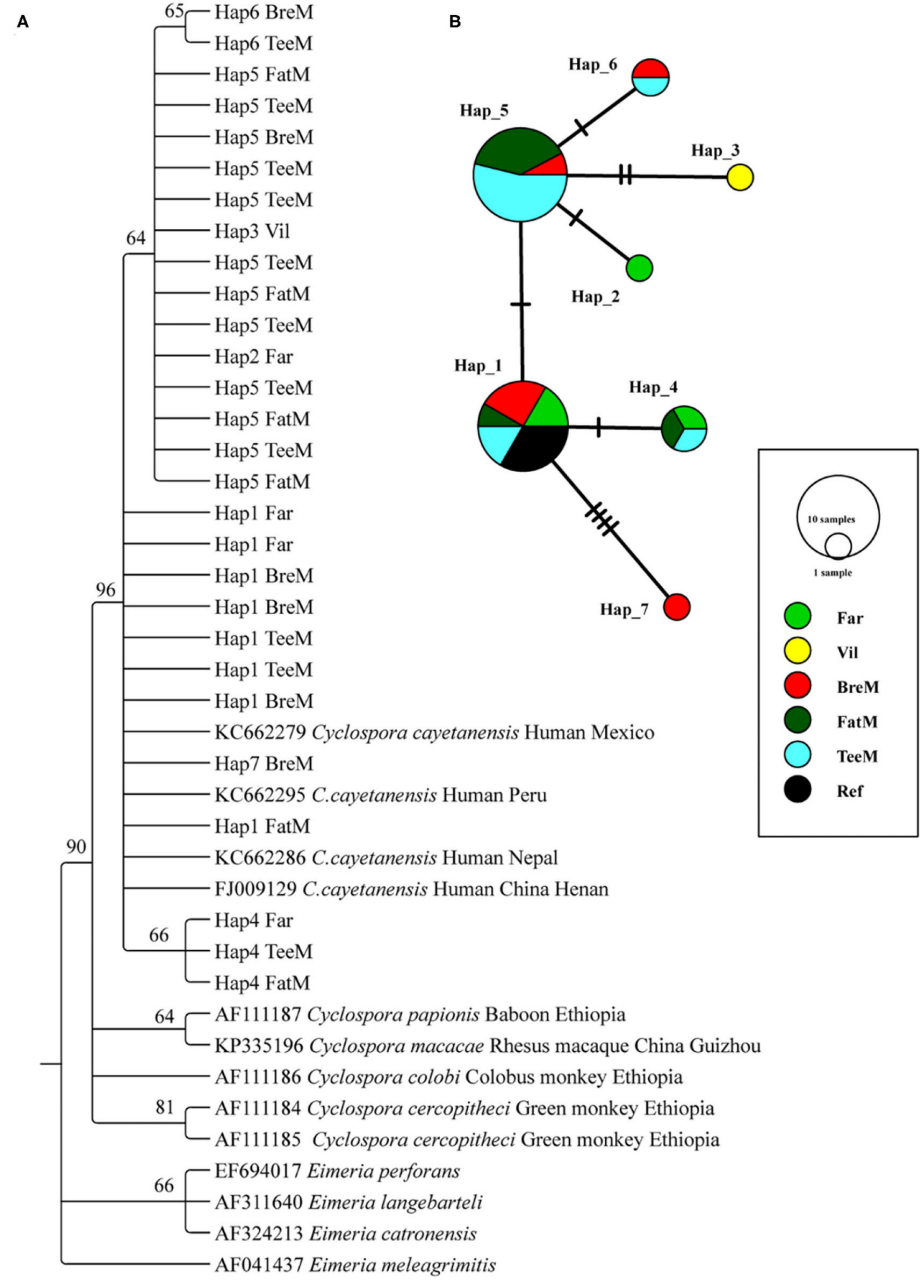
Phylogenetic tree constructed using neighbor-joining (NJ) method and haplotype analysis network of *Cyclospora cayetanensis* based on 423 bp sequence of the partial *SSU rRNA* gene. **(A)** The phylogenetic analysis tree was constructed using the Kimura two-parameter model with 100 bootstrap replications. Eleven haplotype sequences were used in tree construction. **(B)** All the 28 sequences in this study and 4 published sequences of *C. cayetanensis* cluster were used to generate haplotype data. The network was constructed using TCS Network algorithm. Various colors represent different groups and the sizes of the circle represent the numbers of haplotypes. Far, facility worker; Vil, villager; BreM, breeding macaques; FatM, fattening macaques; TeeM, teenage macaques; AduM, adult male macaques; Ref, reference published sequences.

A total of 23 and 5 partial *SSU rRNA* gene sequences of *C. cayetanensis* were obtained from macaques and humans, respectively (Figure 2A). The phylogenetic tree was reconstructed using all the sequences after trimming, and *C. cayetanensis* from the previous studies (24) were used as the reference sequences. All the *Cyclospora* species identified in this study belonged to *C. cayetanensis* cluster, which differed from the other *Cyclospora* spp. found in the NHPs at 96% bootstrap values. Moreover, the three sub-clusters presented in the *C. cayetanensis* cluster were consistent with the results from the analysis of haplotype networks (Figure 2A). A total of seven haplotypes were shown in the network analysis. Four haplotypes (Hap1–4) were detected in the human samples, while five haplotypes (Hap1 and Hap4–7) were detected in macaques. Hap5 was the most common haplotype, accounting for 52.2% of all the haplotypes in macaques (Supplementary Table S2). Moreover, Hap1 was shared among humans, macaques, and the reference sequence from previous studies (Figure 2B).

## Discussion

Our study documented a relatively high prevalence of enteric parasites in NHPs in the Guangxi district, southwest China. Four pathogenic intestinal protozoan species were identified and analyzed. More than 36% of the macaques (in 310 tested macaque samples) in laboratory animal facilities were infected with at least one intestinal protozoa. Among them, the infection rate of *E. bieneusi* was 7.7%. This result is similar to other studies performed previously at the laboratory NHP facility or wild fields (20, 25, 29). For example, the previous studies indicated that *E. bieneusi* was the most common intestinal protozoan infection in the NHPs (29, 30), and Ye et al. revealed the prevalence rate of 18.53% for *E. bieneusi* in a laboratory animal facility in Guangxi (29). Another study of 197 fresh fecal samples obtained from eight NHP species in the Qinling Mountains revealed an *E. bieneusi* prevalence of 12.7% (20). However, we should notice that the composition of the group size and population structure may affect prevalence results.

Three species of zoonotic *Cryptosporidium* spp., including *C. parvum, C. hominis*, and *C. andersoni*, were detected in this study. The first two accounted for over 90% of the infections in the specified host (11). In previous studies, the most prevalent species infecting the NHPs was *C. hominis* (25), inconsistent with the results of this study. In our study, the positive rate of *Cryptosporidium* spp. was 14.4%, much higher than 0.5% in the study of Ye et al. (29), 0.7% (19/2,660) in the study of Karim et al. (31), and 3.0% (6/197) in the study of Du et al. (20). We speculated that this might be due to the long-term semi-closed environment in the facility, which led to repeated infections, and the positive rate also increased in the absence of better prevention and control strategies.

*C. cayetanensis* is the only species of the genus *Cyclospora* with strict host adaptation only known to infect humans, which has not been found in any of the animals studied before (2). Notably, the sequence analysis of the *SSU rRNA* gene showed the prevalence of *C. cayetanensis* in NHPs in our study. The overall prevalence of *C. cayetanensis* was 7.4% (23/310) in macaques but not detected in the AduM group. The phylogenetic analysis clearly showed that the sequences from our study differed from the previous characterization of *Cyclospora* spp. in NHPs such as *Cyclospora papionis, Cyclospora cercopitheci, Cyclospora colobi*, and *Cyclospora macacae* (16, 22, 32, 33). Considering the failure to establish the experimental *C. cayetanensis* infection in *M. mulatta* and *M. fascicularis* (34), the question regarding its host specificity is still unanswered. In contrast, one study reported the detection of *Cyclospora* sp. and *C. cayetanensis* in three *Pan troglodytes* (13.6%) and nine (9.3%) *M. fascicularis* in Europe (23). The results of haplotype network analysis in this study showed two common haplotypes found in macaques. The Hap5 was the most common haplotype (52.2%) observed in macaques only, but not in humans. Moreover, the sequence of Hap5 had only one single nucleotide difference from Hap1 at position 69 in the 423-bp partial *SSU rRNA* gene (T → C). It was speculated that this transition might result in the transmission from humans to macaques, and then the parasites circulate effectively in the population of macaques, forming their unique haplotypes. Since the Hap5 was not detected in humans, this raised a worrying question of whether this haplotype is transmissible to humans or not.

NHPs are commonly infected with *G. intestinalis*. However, understanding of its epidemiology remains incomplete and is primarily based on microscopical analysis. Giardia cysts were found in 31% of macaque samples (belonging to assemblage B, by sequencing PCR products) in wild rhesus macaques (*M. mulatta*) living in urban and semi-rural North-West India. Meanwhile, a molecular prevalence of *Giardia* spp. was recorded about 1% in bonnet macaque in another Indian study (35). We also noticed a total *G. duodenalis* infection rate of 7% among 200 long-tailed macaques' fecal samples (*M. fascicularis*) in Thailand in 2004 (36). Karim et al. (30) investigated the intestinal protozoa from 26 NHP species in China and found that the positive rate of *G. duodenum* was 2.2% (30/1,386).

Similarly, *G. intestinalis* infection was noticed in 7.1% of the macaques under our investigation, and there was no significant difference in infetion rate among different groups. Although, the percent prevalence of *Giardia* spp. varied across the above studies. However, these facts indicate that these animals may potentially contribute to the transmission of human giardiasis. The sequence analysis of the *ITS* gene identified five known genotypes (PigEBITS7, Peru8, Henan V, D, and CM1) in *E. bieneusi* in macaques, which were previously reported in humans (21). The genotype CM1 was the most prevalent genotype (9/24) identified in the stool samples of macaques. Similar results have also been reported in the 23 NHP species from five provinces of China, which considered the CM1, D, and IV as the dominant genotypes (30). All these genotypes were also the members of the group 1 cluster, thereby indicating the zoonotic potential. In our study, the most prevalent *E. bieneusi* genotype in humans was genotype D, with 57.1% (4/7) prevalence. In addition, it was worth noticing that the genotype CM1 was only identified in the facility workers but not in the villagers, which implied that this might be attributed to their direct contact with macaques.

In this study, we investigated the prevalence of intestinal protozoans and related risk factors in both humans and macaques. In two different human populations, the OR of facility workers was higher as compared to the villagers. This result was not surprising since the workers have already been exposed to several risk factors, including direct contact with animals and possible soil, water, and other environmental contamination. From this perspective, health awareness and education among the facility workers are required to prevent and control potential intestinal protozoan infections in this and other similar facilities (7, 37). In the different macaque groups, the OR in the FatM group was higher than that in the AduM group, which may be attributed to their nutrition support or high feeding density. These findings could also provide guidance for the veterinarian to pay attention to animal health caused by such kind of potential risk factors.

Understanding the pathogenic intestinal protozoa infection among humans and animals is a prerequisite for comprehending pathogen and host interactions and dynamics of interspecies dissemination. Our findings added some valuable shreds of evidence indispensable for comprehensive strategy making to reduce zoonosis. However, there are several limitations in the present study. First, the small sample number is a limitation of the present study, especially the villages in this area, decreasing the statistical credibility of finding divergence between different groups. It is necessary to repeat these tests with more samples and in more NHP facilities in future studies. Second, intestinal protozoa are among the most relevant pathogens of foodborne or waterborne disease. Environmental factors such as water supply infection test is overlooked in our study. There is a possibility that unsuspected ecological factors contribute to the risk associated with infection. One Health concept initiative needs to be well established to identify relevant environmental and socio-economic factors that may provide a unique perspective to zoonosis and reverse zoonosis interventions. Third, unfortunately, we failed to amplify the *gp60* gene, the most commonly used genetic locus for subtyping. The molecular subtyping of intestinal protozoa could offer beneficial insights into disease pathogenesis and interspecies transmission. Therefore, it is necessary to confirm the exhaustive information about the subtypes in future investigations.

## Conclusions

This study revealed a relatively high prevalence of intestinal protozoa infections in macaques, facility workers, and villages nearby. The presence of potentially zoonotic intestinal protozoa, in particular the shared haplotypes, could pose health risks between wildlife and humans. Understanding protozoan epidemiology is essential for the health of animals and also for public health. Close contact with wild or captive NPHs is a risk factor for infection, as identified by various studies suggesting that we should limit contact with animal or their excreta. If exposed, we need to take hygienic precautions to mitigate the risk of zoonotic infection and appropriate strategies to control transmission adequately. The utility of One Health frameworks is recommended to characterize infection risk and to offer relevant and comprehensive control strategies in the future.

## Data Availability Statement

The datasets presented in this study can be found in online repositories. The names of the repository/repositories and accession number(s) can be found in the article/Supplementary Material.

## Ethics Statement

The studies involving human participants were reviewed and approved by Ethics Committee of Fudan University. The patients/participants provided their written informed consent to participate in this study. The animal study was reviewed and approved by Ethics Committee of Fudan University.

## Author Contributions

JL, YJR, and HYC conceptualized the study. JL and YJR made significant contributions in the methodology. JL, YJR, and WYH made significant contributions in the investigation. JL and YJR were responsible for writing and preparation of the original draft. XYF and JL were responsible for reviewing and editing the manuscript. XYF and WH supervised the study. All authors contributed to the article and approved the submitted version.

## Funding

This study was supported by the Guangxi Traditional Chinese Medical University Scientific Research Project (XP021059); National Parasitic Resources Center (NPRC-2019-194-30); Key Technology Project of Inner Mongolia Science and Technology Department (2021GG0171).

## Conflict of Interest

The authors declare that the research was conducted in the absence of any commercial or financial relationships that could be construed as a potential conflict of interest.

## Publisher's Note

All claims expressed in this article are solely those of the authors and do not necessarily represent those of their affiliated organizations, or those of the publisher, the editors and the reviewers. Any product that may be evaluated in this article, or claim that may be made by its manufacturer, is not guaranteed or endorsed by the publisher.
