# Supplementary material for: Risk Evaluation of Pathogenic Intestinal Protozoa Infection Among Laboratory Macaques, Animal Facility Workers, and Nearby Villagers From One Health Perspective
Source: Front Vet Sci. 2021 Sep 29;8:696568. doi: 10.3389/fvets.2021.696568 (PMC8511526; doi:10.3389/fvets.2021.696568)
Supplement: Supplementary file 1 [file Table_1.docx]

| Haplotypes | Far* | Vil | Humans | BreM | FatM | TeeM | AduM | Macaques | Genotypes |
| --- | --- | --- | --- | --- | --- | --- | --- | --- | --- |
| Hap1 | 3 | 1 | 4 (57.1) | 1 | 1 | 0 | 2 | 5 (16.7) | D |
| Hap8 | 0 | 0 | 0 | 0 | 1 | 0 | 0 | 1 (4.2) | PigEBITS7 |
| Hap9 | 0 | 0 | 0 | 0 | 0 | 0 | 2 | 2 (8.3) | Peru8 |
| Hap18 | 0 | 0 | 0 | 0 | 0 | 0 | 1 | 1 (4.2) | Henan V |
| Hap19 | 2 | 0 | 2 (28.6) | 3 | 4 | 1 | 1 | 9 (37.5) | CM1 |
| Hap20 | 0 | 0 | 0 | 1 | 0 | 0 | 0 | 1 (4.2) | MEB2 |
| Hap21 | 0 | 0 | 0 | 0 | 1 | 0 | 0 | 1 (4.2) | MEB6 |
| Hap22 | 0 | 0 | 0 | 0 | 2 | 0 | 1 | 3 (12.5) | MEB3 |
| Hap23 | 0 | 0 | 0 | 0 | 0 | 0 | 1 | 1 (4.2) | MEB1 |
| Hap24 | 0 | 0 | 0 | 0 | 1 | 0 | 0 | 1 (4.2) | MEB4 |
| Hap25 | 1 | 0 | 1 (14.3) | 0 | 0 | 0 | 0 | 0 | MEB5 |
| Total | 6 (85.7) | 1 (14.3) | 7 | 5 (20.8) | 10 (41.7) | 1 (4.2) | 8 (33.3) | 24 |  |

Table S1: Haplotypes generated from the *Enterocytozoon bieneusi* positive samples of humans and macaques

*Abbreviations meaning: Far = Facility workers; Vil = Villagers; BreM = Breeding Macaques; FatM = Fattening Macaques; TeeM = Teenage Macaques; AduM = Adult Male Macaques.

****** Number of positive specimens (%)
