# Supplementary material for: Risk Evaluation of Pathogenic Intestinal Protozoa Infection Among Laboratory Macaques, Animal Facility Workers, and Nearby Villagers From One Health Perspective
Source: Front Vet Sci. 2021 Sep 29;8:696568. doi: 10.3389/fvets.2021.696568 (PMC8511526; doi:10.3389/fvets.2021.696568)
Supplement: Supplementary file 2 [file Table_2.docx]

| Haplotypes | Far* | Vil | Humans | BreM | FatM | TeeM | Macaques |
| --- | --- | --- | --- | --- | --- | --- | --- |
| Hap1 | 2 | 0 | 2 (40.0)** | 3 | 1 | 2 | 6 (26.1) |
| Hap2 | 1 | 0 | 1 (20.0) | 0 | 0 | 0 | 0 |
| Hap3 | 0 | 1 | 1 (20.0) | 0 | 0 | 0 | 0 |
| Hap4 | 1 | 0 | 1 (20.0) | 0 | 1 | 1 | 2 (8.7) |
| Hap5 | 0 | 0 | 0 | 1 | 4 | 7 | 12 (52.2) |
| Hap6 | 0 | 0 | 0 | 1 | 0 | 1 | 2 (8.7) |
| Hap7 | 0 | 0 | 0 | 0 | 0 | 1 | 1 (4.3) |
| Total | 4 | 1 | 5 | 5 | 6 | 12 | 23 |

Table S2: Haplotypes generated from the *Cyclospora cayetanensis* positive samples of humans and macaques

*Abbreviations meaning: Far = Facility workers; Vil = Villagers; BreM = Breeding Macaques; FatM = Fattening Macaques; TeeM = Teenage Macaques; AduM = Adult Male Macaques.

****** Number of positive specimens (%)
